# Trypsin-mediated enzymatic degradation of type II collagen in the human vitreous

**Published:** 2013-07-20

**Authors:** Mariëlle van Deemter, Roel Kuijer, Hendri Harm Pas, Roelofje Jacoba van der Worp, Johanna Martina Maria Hooymans, Leonoor Inge Los

**Affiliations:** 1 Department of Ophthalmology, University Medical Center Groningen, University of Groningen, Groningen, The Netherlands; 2 Department of Biomedical Engineering, University Medical Center Groningen, University of Groningen, Groningen, The Netherlands; 3 Department of Dermatology, University Medical Center Groningen, University of Groningen, Groningen, The Netherlands; 4 W.J. Kolff institute, Graduate School of Medical Sciences, Groningen, The Netherlands

## Abstract

**Purpose:**

Aging of the vitreous body can result in sight-threatening pathology. One aspect of vitreous aging is liquefaction, which results from the vanishing of collagen fibrils. We investigated the possibility that trypsins are involved in vitreous type II collagen degradation.

**Methods:**

Immunohistochemistry and western blotting were used for detecting and locating trypsin isoforms in the vitreous and retina of human donor eyes. The capability of the retina to produce these trypsins was analyzed with polymerase chain reaction. Whether the different trypsins degraded type II collagen was tested in vitro. The sizes of the in vitro induced type II collagen degradation products were compared to those present in the vitreous of human eyes of different ages.

**Results:**

Trypsin-1 and trypsin-2 were detected in the vitreous. In the retina, messenger ribonucleic acid (mRNA) coding for trypsin-2, -3, and -4 was present. Using immunohistochemistry, trypsin-2 was detected in microglial cells located in the vitreous and the retina. All trypsin isoforms degraded type II collagen and produced degradation products of similar sizes as those present in the vitreous.

**Conclusions:**

Trypsin-1 and trypsin-2 appear to have a function in the degradation of vitreous type II collagen. They could therefore have a role in the development of vitreous liquefaction.

## Introduction

The vitreous body, the largest structure of the eye, is surrounded by the retina and the lens. With aging, the vitreous body is subject to structural changes, such as the formation of liquid-filled spaces (synchysis) and dense condensations (syneresis) [1-3]. In itself, this can give rise to the perception of floaters, which can seriously interfere with visual functioning. Vitreous liquefaction can be followed by the occurrence of a posterior vitreous detachment [4], which is associated with sight-threatening pathologies such as peripheral retinal tears [5-8], retinal detachment [5-7], macular holes and puckers, and intravitreal hemorrhage [5-7]. In addition, studies have demonstrated that a previous vitrectomy results in increased risk of cataract formation and glaucoma development [9-11]. By analogy, it is interesting to speculate that vitreous liquefaction might be causatively related to the formation of age-related cataract and glaucoma. Therefore, identifying causative mechanisms of vitreous liquefaction and finding possible ways to influence this process is important.

The vitreous is a gel consisting of extracellular matrix with the most important structural component heterotypic collagen fibrils, in which type II collagen is predominant [12,13]. Removal of collagen fibrils in vitro leads to liquefaction of the gel [1,2,14]. The spacing between the fibrils is thought to be maintained by type IX collagen, which covers the fibrils [15]. Due to its single chondroitin sulfate chain, type IX collagen is also classified as a proteoglycan. High concentrations of hyaluronan retain large amounts of water: 98% to 99% of the vitreous consists of water [16,17].

In vivo, vitreous liquefaction is probably caused by enzymatic degradation of collagen. Some evidence consists of the presence of collagen fragments near liquefied spaces and the presence of specific type II collagen degradation products formed by active matrix metalloproteinases (MMPs) [18,19]. We previously showed with western blotting that several collagen degradation products could not be accounted for by the action of MMPs [19].

Another suitable candidate enzyme family that could be involved in type II collagen degradation is that of the trypsins. The trypsin family consists of four enzymes, i.e., trypsin-1, -2, -3, and -4, with high-sequence homology and originating from three different genes, protease serine (PRSS),1 (trypsin-1), PRSS2 (trypsin-2), and PRSS3 (trypsin-3 and -4; splice variants). They are secreted as inactive trypsinogens, after which a single cleavage results in the formation of active trypsin. Originally, trypsins were discovered in the pancreas, where trypsin-1 and -2 have functions in the digestion of food, whereas trypsin-3 and -4 are thought to have a role in the degradation of protease inhibitors. Trypsin-1 possesses collagenolytic activity toward type I collagen [20]. Trypsin-2 is a neutral serine protease that directly degrades the triple helix of type II collagen [21-23]. The presence and expression of trypsin-2 have also been found in tissues such as the cornea and synovial tissue [23,24].

We hypothesized that one or more members of the trypsin family could be involved in the enzymatic degradation of type II collagen in the vitreous. Therefore, in this study, we searched for evidence that different trypsin isoforms are present in the vitreous and retina of human eyes, and, if so, what cell types produced these enzymes. To accomplish this, we isolated vitreous bodies from donor eyes and analyzed them for the presence of trypsins with western blotting. Immunohistochemistry was used to identify a possible production site of trypsins. Furthermore, we investigated whether trypsins produce the specific type II collagen degradation products found in the human vitreous by degrading purified type II collagen with recombinant trypsins.

## Methods

### Eyes, vitreous bodies, and retinas

Seventeen human eyes without any known disorder were obtained from the Cornea Bank (Amsterdam, the Netherlands) after the corneas were removed and within 48 h postmortem. Five were used for immunohistochemical analysis, and 12 for extracting trypsins from the vitreous body. Additionally, for RNA analysis, three retinas were isolated at the Cornea Bank as quickly as possible, within 20 h postmortem.

### Isolation of vitreous bodies and concentrating vitreous extracts

Vitreous bodies were isolated from 12 eyes (age range: 37–66 years) by successive removal of the sclera, iris, choroid, lens, and retina. Finally, the tightly interconnected parts of the pars plana of the ciliary body were carefully cleaved off. The isolated vitreous bodies were stored at −80 °C until further use. To permit triplicate experiments, three pools were formed consisting of four vitreous bodies each: P1 (37–40 years), P2 (57–61 years), and P3 (39–66 years). Pools were formed according to the availability of the donor eyes. There was some coincidental overlap in age distribution between the pools. We accepted this, since the purpose of the study was to demonstrate the presence of trypsins in the vitreous and not to analyze potential age-related differences. The vitreous bodies of each pool were centrifuged at 30,000 × *g* for 2 h. The supernatants were collected and stored at −80 °C until analysis. After thawing, vitreous supernatants were filtrated through 50 kDa Amicon Ultra filters (Millipore, Billerica, MA). The flow-through was collected and concentrated 100 times on 10 kDa Amicon Ultra filters. These resulting fractions were used to analyze trypsin-1, -2, -3, and -4 with western blotting, and to analyze the collagenolytic activity of the vitreous itself.

### Identification of trypsin-1, -2, and -3/-4 in human eye sections by immunohistochemistry

Human donor eyes (52–79 years) were fixed overnight in 2% paraformaldehyde in phosphate buffered saline (PBS, NaCl 9 g/l, KH_2_PO_4_ 144 mg/l, Na_2_HPO_4_-7H_2_O 795 mg/l; Invitrogen, Grand Island, NY). After washing in PBS, the samples were dehydrated in a series of graded ethanol solutions, incubated in xylol, and embedded in paraffin. The paraffin-embedded human pancreas and human liver were kindly supplied by the Department of Pathology (UMCG, Groningen, the Netherlands) and served as positive controls.

Sagittal sections of 5 µm (for fluorescent double labeling) or 3 µm (serial sectioning for peroxidase staining), mounted on slides and dried overnight at 37 °C, were dewaxed and rehydrated. Antigen retrieval was performed by incubation with 0.01% type XXIV protease (Sigma-Aldrich, St. Louis, MO) for 30 min at room temperature (RT). The sections were stained with one of the following primary antibodies: sheep-antitrypsin-1/2/3 (recognizes all trypsin isoforms), mouse-antitrypsin-1, mouse-antitrypsin-2, rat-antitrypsin-3 (R&D Systems, Minneapolis, MN) or goat-anti-Iba-1 (Abcam, Cambridge, UK; recognizes microglia), all diluted 1:50 in PBS+1% bovine serum albumin (BSA) for 1 h at RT. The mature and pro forms of the trypsins are recognized by the trypsin antibodies. The sheep-antitrypsin-1/2/3 and rat-antitrypsin-3 antibodies do not distinguish between trypsin-3 and trypsin-4. Therefore, from now on we refer to them as sheep-antitrypsin-1/2/3/4 and rat-antitrypsin-3/4. Sections treated similarly but with the primary antibody omitted served as negative controls. Next, the 3 µm serial sections were incubated in PBS, 0.1% H_2_O_2_ for 15 min, followed by washing in PBS. The secondary peroxidase-conjugated antibodies were rabbit-antimouse immunoglobulin or swine-antirabbit immunoglobulin (Dako, Carpinteria, CA), depending on the primary antibody used, diluted 1:50 in PBS, 1% BSA, and 2% human serum. Sections were incubated with antibodies for 45 min at RT, stained with 3-amino-9-ethylcarbazole (Sigma-Aldrich), counterstained with hematoxylin, and sealed with a coverslip. For the fluorescent double labeling, secondary antibodies were fluorescein isothiocyanate–labeled rabbit-antigoat immunoglobulin and tetramethylrhodamine isothiocyanate–labeled rabbit-antimouse immunoglobulin, diluted 1:100 in 4',6-diamidino-2-phenylindole (DAPI), containing 1% BSA and 2% human serum. The fluorescent sections were washed in PBS after which the sections were coverslipped using AF1 antifading reagent (Citifluor, Leicester, England). Analysis was performed on a Leica DMR microscope (Leica Microsystems, Rijswijk, the Netherlands).

### Identification of trypsin-1, -2, -3, and -4 messenger ribonucleic acid in human retina with reverse transcriptase polymerase chain reaction

Three retinas (age range: 70–71 years) were isolated within 6–12 h postmortem and immediately immersed in the lysis buffer of the Invisorb Spin Cell RNA mini kit (Invitek, Berlin, Germany), frozen at −20 °C, and transported to the laboratory in dry ice. Total RNA was isolated from human donor retinas according to the manufacturer’s instructions. RNA concentration and purity were determined with A_260_/A_280_ spectrophotometry (NanoDrop, Isogen-Lifescience, Maarssen, the Netherlands). One microgram of RNA was reverse transcribed using the iScript cDNA Synthesis Kit (Bio-Rad, Hercules, CA). The cDNA was diluted 1:20 with diethylpyrocarbonate-water.

The PCR reaction mixture consisted of 12.5 μl PCR Master Mix (2x; Fermentas, Burlington, Canada), 10.5 μl H_2_O, 1 μl primer set, and 1 μl cDNA. Pancreas cDNA (Applied Biosystems/Ambion, Austin, TX) was used as a positive control for the genes of interest. Primers were developed using the Primer-3 software on the NCBI BLAST website (Table 1) and obtained from Biolegio bv (Nijmegen, the Netherlands). The PCR program was 4 min at 92 °C, followed by 40 cycles of 30 s 95 °C, 20 s 60 °C (for trypsinogen-1, -2, and -4 and glyceraldehyde-3-phosphate dehydrogenase) or 68 °C (for trypsinogen-3) and 20 s 72 °C. The final step was 7 min at 72 °C. PCR products were analyzed on a 2% agarose gel. For each gene, the identity of the PCR product was confirmed with sequencing (Baseclear, Leiden, the Netherlands).

**Table 1:** Primers used in the RT–PCR analysis.

**Primer**	**Sequence**	**Amplicon size (bp)**
PRSS1 (Trypsin-1)	Fw. 5′-TCTGGCGCCGACTACCCAGAC-3′	220
	Rv. 5′-TCTGGGCACAGCCATCACCC-3′	
PRSS2 (Trypsin-2)	Fw. 5′-CTACAAGTCCCGCATCCA-3′	132
	Rv. 5′-GATGTCATTGTCCAGAGTCC-3′	
PRSS3 (Trypsin-3)	Fw. 5′GGCGCTGGGCACAGTTGCTG-3′	136
	Rv. 5′-GCTGATGAGGGAGCCACCGC-3′	
PRSS3 (Trypsin-4)	Fw. 5′-ATTCCTGCCAGCGTGACTC-3′	135
	Rv. 5′-ATCCAGTCCACATAGTTGTAGACC-3′	
GAPDH	Fw. 5′-CCCACTCCTCCACCTTTGA-3′	76
	Rv. 5′-CATACCAGGAAATGAGCTTGACAA-3′	

### Type II collagen degradation by recombinant human trypsin-1, -2, and -3

Purified type II collagen from human cartilage, kindly donated by Prof. Dr. R.A. Bank (UMCG), was dissolved in 0.5 M acetic acid to a concentration of 1 mg/ml, and washed three times on a 100 kDa Amicon Ultra filter with TCNB (50 mM Tris, 10 mM CaCl_2_, 150 mM NaCl, 0.05% Brij-35, pH 7.5). Recombinant human trypsin-1, -2, and -3 (R&D systems) were activated according to the supplier’s instructions with some adaptations. In short, recombinant human (rh-) enterokinase (100 µg/ml, R&D systems) was mixed with an equal volume of thermolysin (3.16 µg/ml, R&D systems) and incubated for 30 min at 37 °C, after which thermolysin was inactivated by the addition of 10 mM phosphoramidon. The activated enterokinase was diluted in TCNB to concentrations of 2, 0.4, or 0.8 µg/ml and added to trypsin-1, -2, or -3 in a 1:9 ratio, respectively. This mixture was incubated for 30 min at room temperature and added to the type II collagen in TCNB in a 1:2 ratio, followed by another incubation for 5 h at room temperature. The negative control samples consisted of equal amounts of type II collagen that were incubated for 0 or 5 h in TCNB without enzyme, or 5 h in TCNB with activated recombinant human enterokinase (2 µg/ml). Furthermore, a control sample was made in which activated trypsin (Invitrogen, Cat No. 25050) was blocked by simultaneously adding 1 mM phenylmethylsulfonyl fluoride (PMSF; Sigma Aldrich) to type II collagen. Western blotting was used to analyze the type II collagen degradation products.

### Type II collagen degradation by vitreous extracts

Rh-enterokinase (R&D Systems) was activated according to the supplier’s instructions, and added to a vitreous extract (P3) in a concentration of 2 µg/µl. The mixture was incubated for 30 min at 37 °C. Then, purified human type II collagen, dissolved and washed as described above, was incubated with either the rh-enterokinase-treated vitreous extract, or untreated vitreous extract for 5 h at room temperature. The resulting collagen degradation was analyzed with western blotting.

### Identification of trypsin-1, -2, and -3/4 in vitreous extracts and trypsin-generated type II collagen degradation products with western blotting

Western blots of vitreous extracts and trypsin digests of type II collagen (diluted 1:3 with 4X concentrated sample buffer) were prepared from 10% polyacrylamide slabgels, then blocked with Tris buffered saline (TBS) supplemented with 2% skimmed milk, and stained overnight with one of the following antibodies: mouse-antitrypsin-1 or -2, rat-antitrypsin-3/4 (R&D Systems), mouse-antitype II collagen (ab-3; LabVision, Fremont, CA), all diluted at 1:500. The secondary antibody was goat-antimouse immunoglobulin (1:1000 in TBS, 0.05% Tween-20 (TBST); Jackson ImmunoResearch Laboratories, Carpinteria, CA), which also recognizes rat immunoglobulin. As the tertiary antibody, alkaline phosphatase (AP) conjugated rabbit-antigoat immunoglobulin (1:1,000 in TBST; Jackson ImmunoResearch Laboratories) was used. Protein bands were visualized by incubating the membrane in nitroblue tetrazolium/5-bromo-4-chloro-3-indolyl phosphate solution (Bio-Rad).

## Results

### Trypsin isoforms in the vitreous

**Figure 1:**
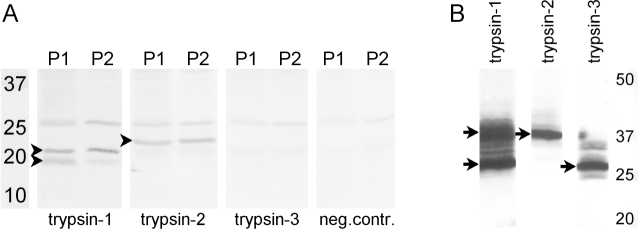
Western blots of vitreous extracts and control tissues incubated with trypsin-1, -2, or -3/4 antibodies. Panel **A** is concentrated extracts of two vitreous pools (P1 and P2) that were analyzed for the presence of the different trypsin isoforms. Trypsin-1 was present as a double band and trypsin-2 as a single band (arrowheads). No specific staining was seen after the antibody against trypsin-3 was used. The left lane contains the molecular weight marker. Panel **B** shows the positive controls for detecting trypsin, in which recombinant human trypsinogen-1, -2, and -3 were used. Trypsin-1 is present as a double band as well and trypsin-2 and -3 as single bands (arrows). The sizes of all bands were higher than those detected in the vitreous samples. In all cases, some lighter-stained bands were present. The antibodies used were mouse-antitrypsin-1, mouse-antitrypsin-2, and rat-antitrypsin-3/4. The right lane contains the molecular weight marker.

Vitreous extracts were analyzed with western blotting for the presence of the trypsin isoforms -1, -2, and -3/4 (P1 and P2 are shown in Figure 1). All vitreous pools showed bands stained by the antitrypsin-1 and trypsin-2 antibodies at approximately 22 kDa and 23 kDa, respectively. These molecular weights were somewhat lower than those found for recombinant human trypsin-1 and -2, which, as stated on the supplier’s datasheet, had a somewhat higher molecular weight than predicted from the amino acid sequence. This is possibly caused by different posttranscriptional modifications. Trypsin-1 was in the vitreous and in the recombinant samples present as a double band. The reason is unknown to us, but again may have to do with posttranscriptional modifications. Trypsin-3/4 was not detected.

### Immunohistochemistry

**Figure 2:**
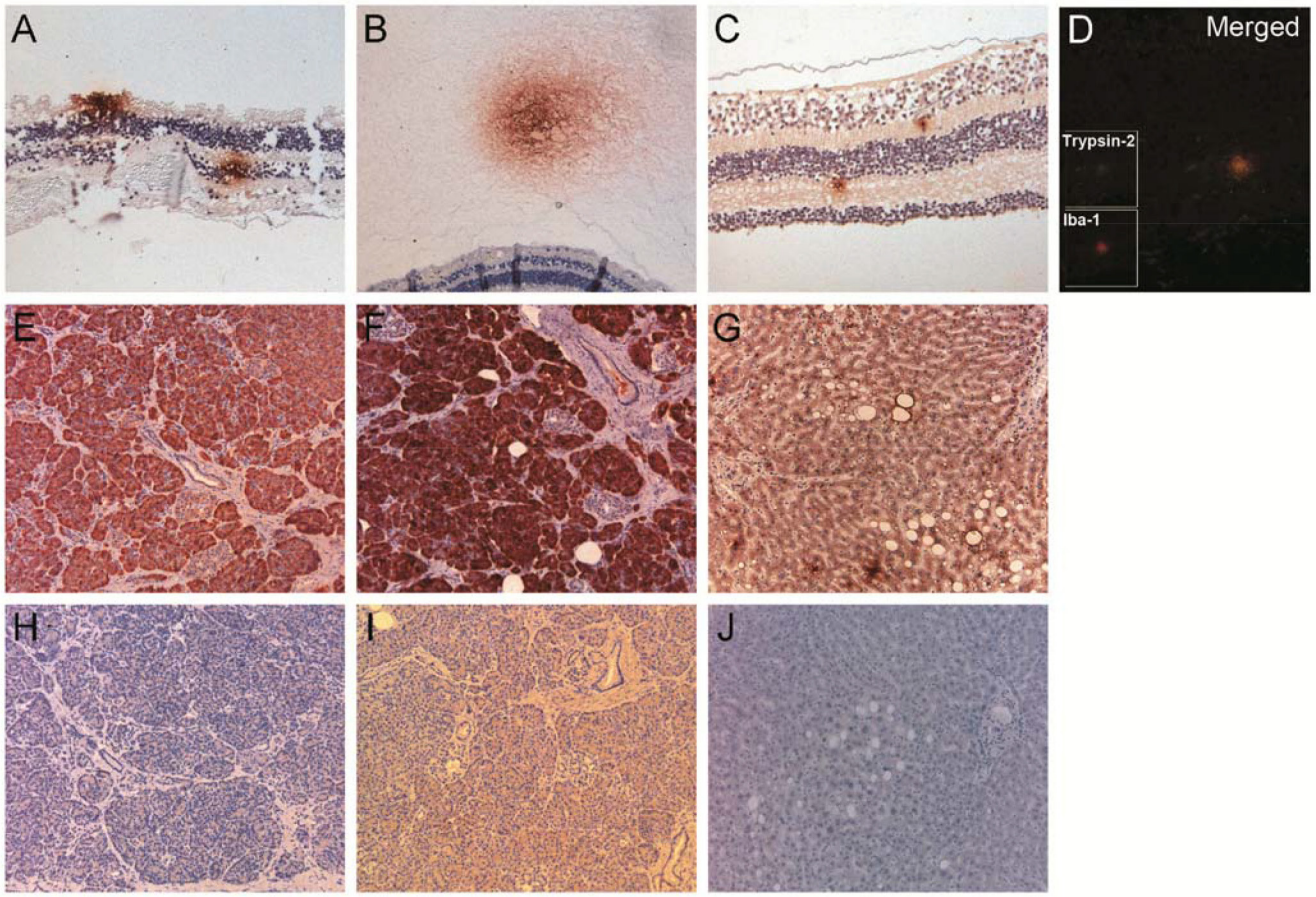
Immunohistochemical detection of the presence and location of trypsins. Using the antibody sheep-antitrypsin-1/2/3/4, star-shaped structures were found in the retina (**A**) and in the vitreous (**B**). Similar structures were discovered using the mouse-antitrypsin-2 antibody (**C**). Double labeling showed colocalization of trypsin-2 (green) and Iba-1 (red), a microglia marker, in the vitreous (**D**) and retina (not shown). The positive control pancreas was stained after the antibodies sheep-antitrypsin-1/2/3/4 (**E**) and mouse-antitrypsin-1 (**F**) were used. The liver showed staining when the mouse-antitrypsin-2 antibody was used (**G**). The negative controls, lacking the primary antibody, showed no staining (**H**–**J**). Panel **H** is the negative control for sheep-antitrypsin-1/2/3/4, **I** is the negative control for mouse-antitrypsin-1, and **J** is the negative control for mouse-antitrypsin-2.

By using the sheep-antitrypsin antibody, which recognizes all trypsin isoforms (i.e., 1, 2, and 3/4), star-shaped structures were found in the retina as well as in the vitreous (Figure 2A,B). Similar structures, but fewer in number, were found after staining with the mouse-antitrypsin-2 antibody (Figure 2C). Because of their location, size, and shape and the low abundance of the detected structures, we suspected these structures were microglial cells. To confirm this, we performed fluorescent double labeling using antibodies against the different trypsin isoforms and the microglial marker Iba-1. Trypsin-2 and Iba-1 colocalized in the star-shaped structures (Figure 2D). Trypsin-1 and trypsin-3/4 were not detected in the retina or vitreous. The functionality of the trypsin antibodies was tested on the pancreas and liver as positive controls. Pancreas staining confirmed functional antibodies against trypsin-1/2/3/4 and trypsin-1 (Figure 2E,F). Liver staining confirmed the functionality of the trypsin-2 antibody (Figure 2G). No staining was observed in any of the controls when the trypsin-3/4 antibody was used (not shown). The negative control sections did not stain (Figure 2H,I,J).

### Reverse transcriptase polymerase chain reaction

**Figure 3:**
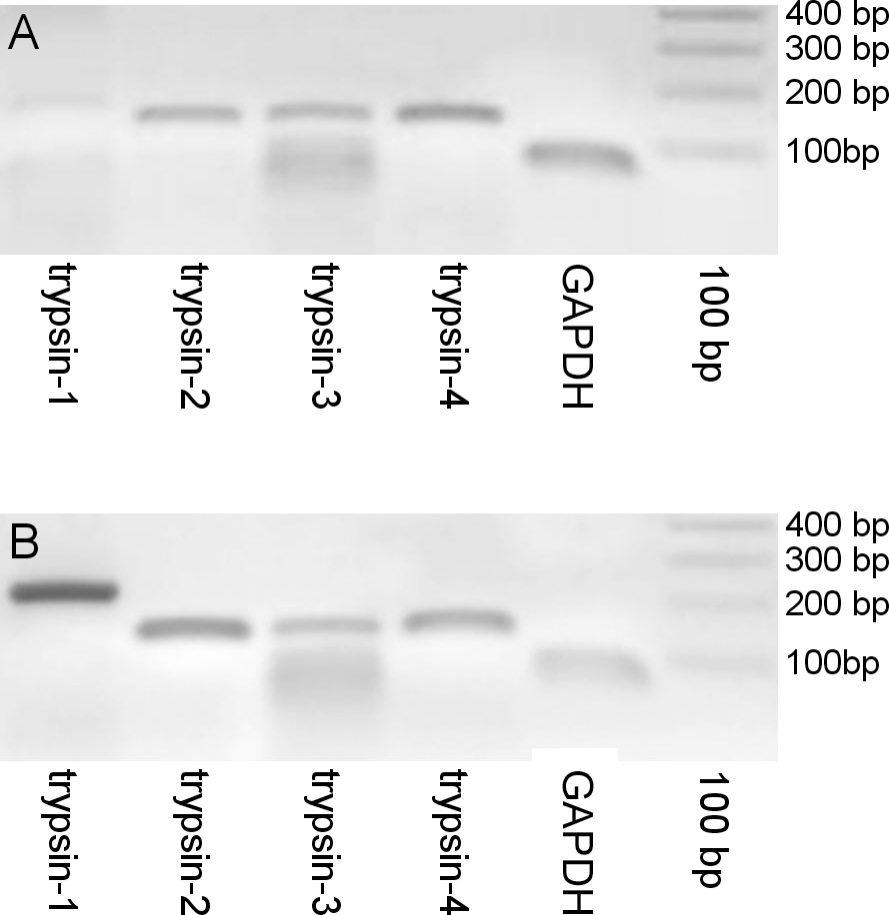
Messenger ribonucleic acid expression of trypsinogen isoforms in the retina and pancreas. **A**: Messenger ribonucleic acid (mRNA) coding for trypsinogen-2, -3, and -4 was found in all retinas with reverse transcriptase polymerase chain reaction (RT–PCR). No trypsinogen-1 coding mRNA was found in any retina. **B**: Testing of the primers on pancreas complementary deoxynucleic acid (cDNA), which served as positive control, showed that all primers produced products of correct size (**A** and **B**). The housekeeping gene glyceraldehyde-3-phosphate dehydrogenase (GAPDH) was used as a control to confirm the presence of RNA in the samples. The 100 bp DNA marker is shown on the right side of the gels.

Three human retinas were tested for the presence of mRNA coding for the different trypsinogen isoforms (the results for one retina, of a 70-year-old female, are shown in Figure 3A). All retinas contained mRNA coding for trypsinogen-2, -3 and -4. Trypsinogen-1 mRNA was not found in any retina. The positive control pancreas showed that all primers used resulted in a gene product of the correct size (Figure 3B).

### Type II collagen degradation with recombinant human trypsin isoforms

**Figure 4:**
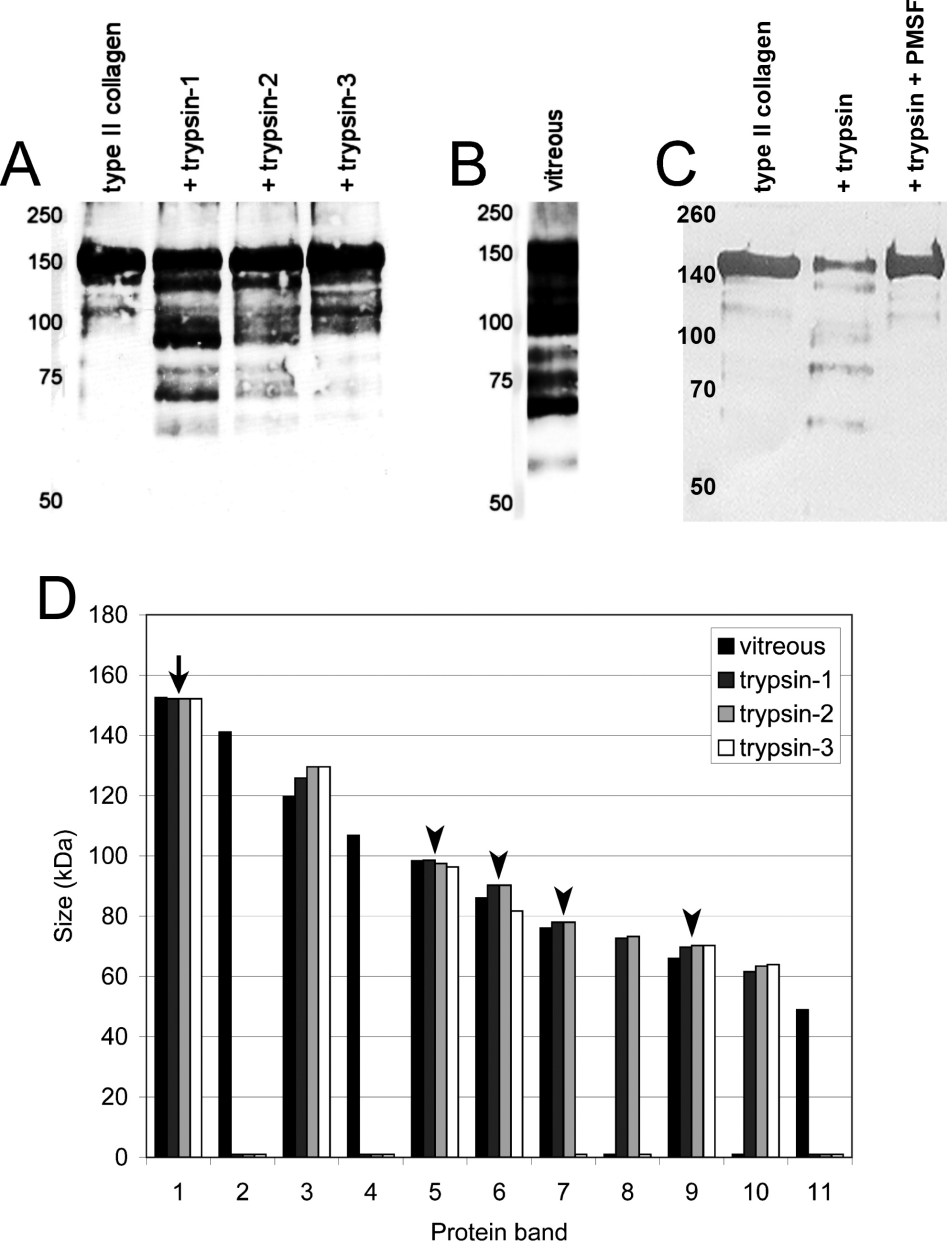
Type II collagen degradation by recombinant human trypsins. Panel **A** shows uncleaved type II collagen from cartilage (left lane) and the degradation patterns after incubation with trypsin-1, -2, and -3 (other lanes). Panel **B** shows the type II collagen degradation pattern of the human vitreous [19]. Inhibition of collagenolytic trypsin activity by phenylmethylsulfonyl fluoride (PMSF) is shown in panel **C**. The left lanes of all western blots show the molecular weight marker. In panel **D**, all bands found in panel **A** and **B** were numbered according to decreasing size and depicted in a bar chart. Protein band 1 (arrow) represents intact type II collagen. Of protein bands 5, 6, 7, and 9 (arrowheads), the sizes of the in vitro formed type II collagen degradation products are comparable with those found in the vitreous. Protein band 3 (asterisk) in the vitreous could be a trypsin degradation product, although it does seem to be a bit small.

When intact type II collagen was incubated with activated trypsin-1, -2, or -3, western blot analysis revealed similar degradation profiles for the three trypsins (Figure 4A). The protein bands were numbered, starting with the largest protein of interest, i.e., intact type II collagen, after which the sizes of these bands were calculated and compared to those found in the human vitreous (Figure 4B) by using a bar diagram (Figure 4D). At least four collagen fragments, of approximately 98, 90, 78, and 70 kDa, were of a size comparable to those found in the vitreous. A fifth fragment, of approximately 125–130 kDa, could possibly be related to the 120 kDa band found in the vitreous, although in that case the vitreous band had probably been subject to additional degradation by another enzyme, resulting in a slightly decreased fragment size. Figure 4C shows that no degradation products were formed if activated trypsin was inhibited with phenylmethylsulfonyl fluoride. In addition, no degradation products formed if type II collagen was incubated without enzyme or with only activated enterokinase (not shown). Incubation of intact type II collagen with 100X concentrated vitreous extracts for 5 h with or without activation of the trypsins in the extract did not result in degradation of the collagen (results not shown).

## Discussion

In the present study, we show with western blotting that trypsin-1 and -2 are present in the vitreous (Figure 1). Furthermore, we demonstrated that trypsin-1, -2, and -3 produced type II collagen degradation products of sizes comparable to those found in the vitreous (Figure 4). PCR analysis showed that mRNA coding for trypsin-2, -3, and -4 is present in the retina (Figure 3), and immunohistochemical analysis indicated that microglial cells in the retina and the vitreous are the likely source of trypsins (Figure 2). These results provide a plausible explanation for the presence of type II collagen degradation products that previously have been found in the human vitreous [19]. The continuous presence of type II collagen degradation products suggests that enzymatic degradation is an ongoing and slow process. Accordingly, considering that collagen degradation takes place continuously throughout life, enzymatic degradation could be involved in the development of age-related vitreous liquefaction. This is in line with previous findings of collagen fragments in the vicinity of liquefied spaces in the vitreous, which suggests that collagen degradation could be linked with vitreous liquefaction [18].

In the in vitro degradation experiments of type II collagen by trypsins, we needed high concentrations of trypsins to obtain degradation products. Similar results were reported in previous studies on trypsin digestion of intact fibrillar collagen, in which a large molar excess of trypsin had to be used, demonstrating that the actual degradation process is relatively inefficient [20]. Low vitreous trypsin concentrations and, moreover, the presence of several serine-protease inhibitors [25-27], probably explain why our concentrated vitreous extracts did not result in visible degradation of type II collagen. Furthermore, the low solubility of the collagen fibrils at neutral pH will probably result in decreased efficiency of trypsin-induced type II collagen degradation. Compared to the in vivo situation, in which protein degradation is continuously present over life, our experiments lasted for a relatively short time, possibly too short to see an effect.

The fact that trypsin-induced type II collagen degradation products are present in the vitreous, despite trypsin’s apparent inefficiency and the presence of trypsin inhibitors, might be explained by the increased focal trypsin concentrations within and near the microglial cells. Microglial cells are present in the vitreous matrix, but are apparent only in immunohistochemical specimens when specific dyes are used. In histological sections stained with toluidine blue alone, they would not stand out because of their ramified appearance, which is similar to the structure of the surrounding vitreous matrix. This probably also explains why in a previous study no cells were observed near collagen fibril fragments, which led to the conclusion that cells were likely not directly involved in vitreous collagen degradation [18].

Microglial cells can be activated to produce enzymes by oxidative stress [28,29]. In the retina, the highest cellular activity and the maximum amount of incoming light are found at the macula [30,31]. Therefore, most oxidative stress can be expected at this site. Interestingly, vitreous liquefaction is first seen near the macula [32,33]. This supports the hypothesis that microglia-mediated trypsin-induced collagen degradation is likely a cause of vitreous liquefaction. The most important function of microglia is detecting and clearing dead cells and infectious agents [34], so whether collagen degradation is a primary function or a secondary side effect is unknown.

PCR analysis provided further evidence that trypsin-2, -3, and -4 are likely produced by retinal cells. The presence of trypsin mRNA and the proteins themselves colocalizing with microglial cells indicates that the trypsins apparently have a local function instead of having leaked out from the blood into the retina and vitreous. Such leaking, possibly caused by postmortem breakdown of the blood–retinal barrier, could be the cause of the presence of trypsin-1, as it was found only in the vitreous, and its presence and source could not be confirmed with immunohistochemistry or PCR analysis. Apart from degrading collagens, trypsins could also be involved in the weakening of the vitreoretinal adhesion, thus resulting in the development of a posterior vitreous detachment. Remarkably, in contrast to trypsin-1 and -2, western blotting did not detect trypsin-3/4 in the vitreous. However, in a proteomic study, which used a more sensitive method than western blotting, trypsin-4 was found in the vitreous [35]. Unfortunately, the trypsin-3/4 antibody was not suitable for immunohistochemistry, so the possible location of trypsin-3/4 could not be detected.

The finding that proteases that cleave type II collagen rather inefficiently appear to have an active role in vivo sheds new light on the process of collagen turnover in general. Until now, the collagen triple helix was thought to be resistant to cleavage by most proteases apart from specific enzymes, such as the collagenases MMP-1, -8, and -13 [36]. Our results indicate that other enzymes can contribute to collagen degradation. In addition, in cartilage trypsin-induced type II collagen degradation products have been reported [23].

A few caveats must be mentioned. All investigations were performed post mortem. Thus, we cannot know for certain whether trypsins and microglia are active in the living eye, or that this is the result of some postmortem effect (e.g., autolysis or breakdown of the blood–retinal barrier). However, the efficiency of collagenolysis by trypsins is so low that, as a postmortem effect, not a high concentration of trypsin-induced breakdown products would be expected. Furthermore, the enzyme(s) that produce the specific type II collagen degradation products do not necessarily have to be trypsin isoforms. Currently, we do not know if these enzymes are present in an active form or as inactive zymogens. Therefore, we cannot exclude that additional enzymes might be present in the vitreous with similar collagenolytic abilities, and future research should not exclude a search for other proteolytic enzymes. In addition, there is the possibility that the collagen digested by trypsins was previously cleaved by one of the collagenolytic MMP-1, -8, or -13. These MMPs cleave the type II collagen triple helix at a specific site three-quarters from the N-terminus, resulting in the typical 3/4 and 1/4 degradation products. However, the formation of the fragment that appears on western blot at the level of about 120–130 kDa cannot be explained by the action of these MMPs, as it is too high to be the 3/4-degradation product, which on our western blots usually appears at the level of about 110 kDa [37]. Identification of the type II collagen cleavage sites would help understand the enzymatic degradation process and the involved enzymes.

In conclusion, our data are accumulating evidence in favor of continuous collagen turnover in the human vitreous. Previously, evidence for collagen degradation [18,19] and for collagen production by adult retinal cells has been presented [38,39]. The estimated vitreous collagen type II half-life is approximately 15 years. Since collagen concentrations remain stable during adulthood, this finding provides further evidence that type II collagen turnover is actively taking place throughout life [37].

We provide further evidence for enzymatic vitreous collagen breakdown and support the hypothesis of vitreous collagen turnover. This process is possibly induced by oxidative stress and mediated by trypsin-producing microglial cells. The collagen-degrading properties of trypsins in non-pathological conditions provide interesting new perspectives on research into the age-related decline of other collagenous tissues.
